# Keratinocytes under Fire of Proinflammatory Cytokines: Bona Fide Innate Immune Cells Involved in the Physiopathology of Chronic Atopic Dermatitis and Psoriasis

**DOI:** 10.1155/2012/718725

**Published:** 2012-11-05

**Authors:** François-Xavier Bernard, Franck Morel, Magalie Camus, Nathalie Pedretti, Christine Barrault, Julien Garnier, Jean-Claude Lecron

**Affiliations:** ^1^Laboratoire Inflammation, Tissus Épithéliaux et Cytokines, UPRES-EA 4331, CHU de Poitiers, Pole Biologie Santé, Université de Poitiers, Bâtiment B36, 1 rue G Bonnet, 86022 Poitiers, France; ^2^BIOalternatives, 1 bis rue des Plantes, 86160 Gençay, France

## Abstract

Cutaneous homeostasis and defenses are maintained by permanent cross-talk among particular epidermal keratinocytes and immune cells residing or recruited in the skin, through the production of cytokines. If required, a coordinated inflammatory response is triggered, relayed by specific cytokines. Due to numerous reasons, troubles in the resolution of this phenomenon could generate a cytokine-mediated vicious circle, promoting skin chronic inflammation, the most common being atopic dermatitis and psoriasis. In this paper, we discuss the biological effects of cytokine on keratinocytes, more particularly on specific or shared cytokines involved in atopic dermatitis or psoriasis. We report and discuss monolayer or 3D in vitro models of keratinocytes stimulated by specific sets of cytokines to mimic atopic dermatitis or psoriasis. IL-22, TNFa, IL-4, and IL-13 combination is able to mimic an “atopic dermatitis like” state. In psoriasis lesions, over expression of IL-17 is observed whereas IL-4 and IL-13 were not detected; the replacement of IL-4 and IL-13 by IL-17 from this mix is able to mimic in vitro a “psoriasis like” status on keratinocytes. We conclude that specific cytokine environment deregulation plays a central role on skin morphology and innate immunity, moving towards specific pathologies and opening the way to new therapeutic strategies.

## 1. Introduction

Skin constitutes the largest physical and chemical barrier against various stresses including pathogens, constituting the first line of defense of the body. Cutaneous homeostasis and defenses are maintained by permanent cross-talk among dermal fibroblasts, epidermal keratinocytes, and cells of the immune system residing or recruited in the skin, through the production of cytokines. Recent years showed that skin appears as a multitasking immune organ, and highlighted the key role of keratinocytes, which are the epithelial cells of epidermis (the outer skin layer in contact with environment), in this protection [1]. Keratinocytes are no longer considered as a passive protection barrier but as true innate immune cells. Indeed, skin represents a very attractive tissue that is a paradigm for studying the cross-talk between innate and adaptive immunity system and an organ.

To be effective for protection against a large panel of injuries, skin requires sensitivity and selectivity to detect a signal of danger, a strong reactivity to develop a rapid response, and efficiency. These requirements need sophisticated interactions between keratinocytes and “sentinel” immune cells infiltrating normal epidermis, that is, T lymphocytes and Langerhans cells [2]. These interactions are conducted by cytokines, maintaining the homeostasis of skin; if required, a coordinated inflammatory response is triggered, relayed by specific cytokines. Due to a number of known or unknown reasons (genetic, environmental, etc.), troubles in the resolution of this phenomenon could generate a cytokine-mediated vicious circle, promoting chronic inflammation. This state is characterized by resident/infiltrating immune cells in the epidermis or dermis, altered differentiation of keratinocytes and the increase of epidermal thickness in some cases. They are the result in particular of an altered dialogue between keratinocytes and activated immune cells, due on a balance of Th subsets, especially Th1, Th2, Th17, and Th22 producing specific sets of cytokines. Various inflammatory responses can be observed, leading to different clinical entities, the most common being atopic dermatitis (AD) and psoriasis. AD affect up to 3% of adults and 25% of children, and psoriasis 2.5% of the world's population [3]. Both are classically considered as two opposing models due to the polarization of the Th response.

Atopic dermatitis has a complex pathogenesis associating inflammatory reactions to epidermal barrier dysfunctions, allowing allergen sensitization. Genetic studies showed the importance of filaggrin in AD [4]. Filaggrin is a protein of the stratum corneum involved in the maintenance of keratin cytoskeleton, the assembly of the cornified envelope, and the water-binding capacities of skin. Inflammatory cytokines are implicated in skin barrier disruption by downregulating the protein expression of the cornified envelop, including filaggrin.

Chronic lesions of AD are characterized by lichenification with skin thickening and hyperplasia of the epidermis.

Plaques of psoriasis are characterized by parakeratosis, but also skin thickening and acanthotic epidermis with abundant dermal mononuclear cell infiltrate. These two inflammatory chronic skin disorders were opposed by the long standing Th1/Th2 paradigm, despite the fact that they share some histological similarities. The discovery of a role for IL-17-producing T helper cells (Th17) in psoriasis and for IL-22 producing (Th22) cells in AD emphasize the complexity of cytokine network involved in the induction and/or maintenance of these disorders.

Our paper is focused on the cytokines associated to AD compared to those in psoriasis and their direct biological effects on keratinocytes. For a general recent review comparing clinical features, immune cells, and therapeutic strategies in AD and psoriasis, refer to Guttman-Yassky et al. [2, 3]. Likewise, review and the experimental studies reported herein are focused on the chronic phases, occulting the description of initiating events and earlier phases of these skin diseases.

We study *in vitro* the respective effects of AD or psoriasis-associated cytokines on parameters of innate immunity and histology using the (normal human epidermal keratinocytes) NHEKs and (reconstituted human epidermis) RHE, and discussed the results in the context of Th polarization. We shall see that the inflammatory skin phenotype is largely the consequence of the effect of cytokines on keratinocytes. We will comment on the view that, if AD and psoriasis were opposed by long standing paradigms, they also have common features.

## 2. Cytokines and Keratinocytes

During the past few years, an increasing number of reports demonstrated that keratinocytes are direct targets for a specific set of cytokines, conducting dramatic changes in their biological properties such as inducing the secretion of chemokines and antimicrobial peptides or modulating the differentiation states or migration capacities. In the 1990s, functional receptors for the classical inflammatory cytokines interleukin (IL)-1 and tumor necrosis factor (TNF) or the Th1-derived interferon (IFN)-*γ* have been described on keratinocytes (review in [5]). More recently, IL-17, IL-21, IL-22, and oncostatin M (OSM) have also been described as important regulators of epidermis functions, and their receptors have been detected on keratinocytes [6–9]. Other cytokines are able to induce skin inflammation in animal models, as do IL-12 or IL-23. However, keratinocytes do not display functional receptors for IL-12 and IL-23 [10], suggesting that other cell types are the targets, and that the effect on keratinocytes is indirect.

As an example, amongst the cytokines of the IL-10 family, IL-22 is the strongest activator of keratinocytes, and to a lesser extent IL-24, followed by IL-20 and IL-19 [11]. Analysis of the gene expression profile induced on NHEK showed that these cytokines upregulate the expression of genes associated with inflammation and innate immunity, such as S100A7-psoriasin and *β*-defensin2 (BD-2); they also downregulate differentiation-associated genes, including CK10, and induce hyperplasia and hypogranulosis of RHE [11, 12] and upregulated CK16, which is associated with suprabasal keratinocyte proliferation.

The simultaneous analysis of a large panel of cytokines demonstrate that a number of them directly target keratinocytes, that is, cytokines of the IL-1 (IL-1*α* and *β*), IL-2 (IL-4, IL-13, IL-21), IL-4 (IL-4, IL-13), IL-6 (IL-6, OSM, IL-31), IL-10 (IL-19, IL-20, IL-22, IL-24), IL-17 (IL-17A and IL-17F), IFN (IFN*α*, IFN*γ*), or TNF (TNF*α*, TNF*β*) family [5]. Note that the biological effect of IL-4 or IL-13 on the inhibition of defensin production is observed only after preactivation of keratinocytes by the IL-4, IL-13, IL-22, and TNF*α* cytokine combination, which enhance IL-13RA2 chain expression as defined below. Regarding IL-21 receptor, IFN*α* and *γ* enhance the IL-2R*γ* chain expression; since IL-21R is also expressed by keratinocytes (unpublished data), it suggest the sensitivity of keratinocytes to IL-21. It has been confirmed by Caruso et al, demonstrating that IL-21 induces *in vitro* the proliferation of isolated keratinocytes [13] and induces Erk phosphorylation (unpublished data). In contrast and in our hands, there is no evidence for a direct effect IL-2, IL-7, IL-9, and IL-15 on keratinocytes [14]. Transcriptomic analysis of the keratinocyte gene expression profile demonstrates that most cytokines active on keratinocytes have overlapping activities, sometimes displaying completely redundant biological properties. The use of shared molecules in intracellular signaling pathways, such as STAT3 in OSM or IL-22 signaling or NF*κ*B in IL-1 or TNF signaling could explain redundancy [6, 7]. Recently, the IL-36 members of the IL-1 family have been reported to induce by an autocrine effect their own secretion and antibacterial peptides production by keratinocytes [15].

Usually upstream of cytokine action during an inflammatory process, TLR engagement activates NF*κ*B pathways, as IL-1 or TNF receptors engagement do, and may have partial redundant activities with these cytokines on keratinocytes. Keratinocytes express TLR 1-6 and 9 [16, 17]. TLR engagement have been described to induce cytokines, such as TSLP [18] and IFN*α* and *β*. Synergistic and/or overlapping biological activities of TLR ligands and cytokines on keratinocytes are very likely, as discussed below for cytokine combinations. Poorly documented up to now, the study of these costimulations could give a more dynamic view of the course of skin inflammation. 

In another hand, powerful synergistic effects on keratinocytes have been also evidenced when cytokines of different families are associated [14]. These cytokines modified the expression of genes associated with inflammation, innate immunity, and differentiation. However, comparative and quantitative analysis demonstrates huge differences of expression induced by specific cytokines. For example, IL-22, as OSM, both via the STAT3 activation pathway induced a strong inhibition of keratinocyte differentiation, as objectived by the decreased expression of filaggrin, loricrin and involucrin and the increase in the overall thickness of RHE [6, 7]. In contrast, the induction of chemokines, S100A7, or BD-2 by IL-22 and OSM is moderated. On the other hand, whereas IL-17, IL-1, and TNF*α* strongly induced chemokines, S100A7, or BD-2 expression, they have weaker effect on keratinocyte differentiation [14, 19]. Nevertheless, it appears that single cytokine stimulation generates a rather limited effect on keratinocytes, namely, a limited number and/or a limited modulated expression of targeted genes.

Since in physiological or physiopathological conditions, tissues are surrounded not by one cytokine but a complex milieu, study of the biological activities of cytokine combinations is of great interest. For example, combination of IL-17A and IFN-*γ* or IL-17A and TNF-*α* results in a synergistic effect on CXCL8 production by keratinocytes [20, 21]. IL-17A and IL-22 synergize in the upregulation of BD-2 and S100A9 production [22, 23]. The association of IL-1*α*, IL-17, IL-22, OSM, and TNF*α* demonstrated a very strong synergy in increasing the expression of inflammatory molecules such as psoriasin/S100A7 or BD-2, or IL-8 *in vitro* by NHEK [14]. When IL-22 is removed of the cytokine mixture, CXCL8 and BD-2 expression is reduced by 30%, whereas the decrease is about 70% after IL-17 removing. In addition, *ex vivo* studies on human skin explants demonstrated upregulation of BD-2, S100A7, and CXCL8 expression in response to the same combination of cytokines. *In vivo* intradermal injection of these five cytokines in mouse increased CXCL1, CXCL2, CXCL3, S100A9, and BD-3 expression, associated with neutrophil infiltration and an early epidermal acanthosis [14] (and submitted).

## 3. Specific and Shared Cytokines in Atopic Dermatitis and Psoriasis

The presence of CD4^+^CCR4^+^ Th2-cells in lesional skin of acute atopic dermatitis patients was described [24]. Th2 secreted predominantly IL-4 and IL-13, which induce both pro- and anti-inflammatory effects depending on the target cell type and on the nature of the receptors expressed. IL-4 and IL-13 inhibit TNF*α* and IFN*γ*-mediated induction of antimicrobial peptides BD-2 and BD-3 by keratinocytes [25–27]. It has been suggested that the increased expression of IL-4 and IL-13 in atopic dermatitis skin may explain the susceptibility to bacterial and viral skin infections by reducing antimicrobial peptide expression [26, 27]. On the other hand, as TLR3 agonists [18], IL-4 or IL-13 synergizes with TNF*α* or IL-1*β* [28] to induce expression of thymic stromal lymphopoietin (TSLP) in keratinocytes and subsequently induces the maturation of CD11c^+^ dendritic cells involved in allergic inflammation. Besides regulation of their production, IL-4 and IL-13 activities can be modified by differential expression of their receptors. Indeed, IL-4 and IL-13 upregulate IL-13R2 expression in keratinocytes [29] whereas IFN*γ* or IL-13, but not IL-4, upregulates expression of IL-13R1 on keratinocytes *in vitro* [30].

IFN*γ*, mainly produced by the Th1 lymphocyte subset and by natural killer cells, is implicated in the regulation of different cellular processes such as antiviral responses, cell growth and differentiation, and immunoregulatory functions [31]. IFN*γ* levels are increased in psoriatic skin, but not in acute AD lesions [32] reflecting an altered Th1/Th2 balance [33–35]. Interestingly, these IFN levels are reduced after antipsoriatic therapy [36, 37]. It is classically admitted that psoriasis is a Th1 disease. However, the coexistence of both IL-4-producing Th2 and IFN-*γ*-producing Th1 cells or Th1 dominance is observed in chronic AD lesion [38].

Several models strengthen the role of the members of the IL-1 family in inflammatory skin diseases. Transgenic mice constitutively expressing IL-1*α* in basal keratinocytes (under the control of the CK14 promoter) develop a spontaneous skin disease characterized by hair loss and inflammatory skin lesions displaying hyperkeratosis, acanthosis, parakeratosis, and a mononuclear cell infiltrate [39]. In addition, IL-1RA deficient BALB/c mice develop arterial inflammation, arthritis, and a localized skin inflammation resembling human psoriasis [40]. Recently, IL-36, *α*, *β*, and *γ*, 3 other members of the IL-1 family, have been found to be expressed in a psoriasis-like animal skin, as well as in the lesions of psoriatic patients [15].

 TNF*α* is a multifunctional cytokine also produced by Th1 cells that mediates inflammation, immune response, and apoptosis. Biological activities are mediated by two distinct cell surface receptors: TNFR1 and TNFR2. Analysis of mice lacking either TNFR1 or TNFR2 has demonstrated that TNFR1 is critical for induction of skin inflammation by TNF*α* [41]. Intradermal injection of TNF*α* leads to skin inflammation with elevated IL-6 production and induction of ICAM-1 expression by keratinocytes [41]. TNF*α* is also involved in the production of antimicrobial peptide such as BD-2 and BD-3 [26, 42]. Extensive analysis of the transcriptional profile of keratinocytes treated with TNF*α* shows activation of innate and adaptive immune responses by inducing a large panel of chemokines that attracts neutrophils, macrophages, and T cells. The fundamental role played by TNF*α* in skin inflammation has been confirmed in mice lacking either IKK2 or I*κ*B*α*, two molecules of the TNF*α*/NF*κ*B signalling pathway. Finally, expression of TNF*α* and TNFR1 is increased in lesional psoriatic compared to normal skin [43]. These *in vitro* and *in vivo* observations are confirmed by numerous clinical studies of successful anti-TNF therapy in psoriasis since the first report in 2000 [44–46]. In contrast, a pilot study on the effect of infliximab on 9 patients with moderate to severe AD was disappointed [47]. Nevertheless, these cytokines do not seem to be involved in hyperplasia of epidermal keratinocytes observed in chronic skin lesions of AD and psoriasis. The hyperplasia and the alteration of barrier-related functions are linked to Th17 and Th22 cell subsets. Regarding the presence of Th17 lymphocytes in lesional psoriatic skin and the critical role of IL-17 in the pathogenesis of psoriasis [2, 8], presence and involvement of Th17 in AD has been evaluated. The percentage of Th17 cells was increased in peripheral blood of AD patients, in correlation with the severity of AD, and dermis IL-17-immunostaining was strongest in acute lesions when compared to the chronic one [48, 49]. Nograles et al. showed that the Th2 microenvironment has a suppressive effect on Th17 pathway by decreasing IL-17 receptor expression [50]. Whatever the case, IL-17A expression in lesional AD is almost unchanged when compared to normal skin, whereas it is increased in psoriasis [8] (unpublished data). In addition, expression of IL-23p19, IL-12p40, but not IL-12p35, is increased in psoriatic skin [51–53] and IL-23p19 expression is associated with dendritic cells infiltrated in the skin [8].

As soon IL-22 has been described to target keratinocytes, it has been observed that the cytokine induces a “psoriasis-like” phenotype on RHE, that is, hyperplasia with a thickening of the spinous layer and a disappearance of the granular layer [6, 11], and that the expression and secretory patterns of IL-22-treated keratinocytes resembled most of the features of psoriatic lesions [54]. Indeed, IL-22 is overexpressed in psoriatic lesions, whereas IL-22R1 and IL-10R2 are expressed at a similar level in psoriatic and healthy skin [37, 55]. T cells infiltrating psoriatic lesions are an important source of IL-22, higher than peripheral T cells from psoriatic patients or controls [55]. Further analysis of T cell infiltrating psoriatic skin showed IL-17 and IL-22 coproduction by Th17 lymphocytes [56, 57]. Innate sources of IL-17 have been also considered in skin. Recently, a dermal ROR*γ*t *γδ* T cells producing IL-17 following exposure to IL-1*β* plus IL-23 have been described [58, 59]. In psoriatic patients, these *γδ* Tcells were greatly increased in affected skin and produced large amounts of IL-17 [59]. Attention could also be paid to ROR*γ*t+, CCR6+, CD4neg, NK1.1neg iNKT cells [60] as a potential source of IL-17 in psoriasis, since these cells have been also described in skin and draining lymph nodes and respond to inflammatory signals in mice [61].

Beside the Th17 cells, a so-called Th22 subpopulation producing IL-22 but nor IL-17 or IFN*γ* has been recently described in circulation and in normal human dermis, expressing CCR6 and the skin-homing receptors CCR4 and CCR10 [62–64]. Th22 clones derived from psoriatic lesional skin have been further described, mostly in the epidermis compartment of the skin [65]. Not specific of psoriasis, Th22 are also increased in lesional skin of atopic dermatitis and a correlation is observed between the number of Th22 cells and disease severity [66]. In the chronic phase of AD, Th22 subset is induced by Langerhans cells [67] along with a Th1 cell response believed to be induced by IDCs. Otherwise, both in murine and human, it has been suspected that non-T cells in skin produce IL-22, such as dendritic cells, NK cells, macrophages, and so forth. [68, 69]. In our hands, IL-22 expression in lesional AD and psoriasis was enhanced when compared to normal skin [55] (submitted results). Interestingly, rare patients with simultaneous occurrence of psoriasis and AD have been recently described [70]. Predominant antigen (Ag)-specific Th1 and Th17 lymphocytes infiltrates psoriatic lesions whereas Th2 infiltrates AD lesions, and IL-22 are detected in both lesions. Taken together, it appears that psoriasis is overall Th1 and Th17 mediated and AD is overall mediated by Th2 and Th22 cells, and that Th1 cells also contribute to the chronic phase of AD. In most cases, psoriasis and AD are mutually exclusive for individuals, but in rare cases can be concomitants, in which case Ag-specific T cells determine the specific pathogenesis [70].

## 4. Modelization of AD versus Psoriasis by Specific Sets of Cytokines

Taking into account (a) the preferential cytokine environment in AD vs psoriatic lesions and (b) those of these cytokines able to target keratinocytes [14], we design *in vitro* experiments aiming to culture NHEK and RHE with an IL-22, TNF*α*, IL-4, and IL-13 mix or an IL-22, TNF*α*, and IL-17 mix, with the objective to, respectively, mimic AD and psoriasis epidermis. In these models, we finally analyzed the effects of the change from IL-17 (Th17, psoriasis) to IL-4/IL-13 (Th2, AD), in the same cytokine background (TNF*α* and IL-22). We hypothesize that these cytokines are not only specific biomarkers of the diseases, but are responsible for the phenotypes and the molecular signatures of these diseases.

**Figure 1:**
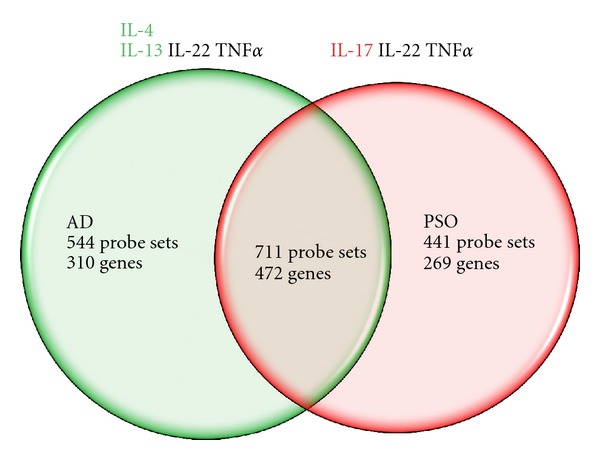
Overall effects of IL-22, TNF*α*, IL-4, and IL-13 combination (AD) or IL-22, TNF*α*, and IL-17 combination (PSO) on the transcriptome of keratinocytes in monolayer culture (NHEK). NHEKs were cultured in duplicate cultures with 10 ng/mL of each cytokines for 24 h and the gene expression profiles were analysed using Affymetrix hU219 chips. The number of significantly modulated (±2-fold the control) genes is indicated for each treatment (average of duplicate analysis) as well as the number of genes modulated by the two treatments.

The overall response of human keratinocyte monolayers (NHEK) to defined cytokines aiming to mimic AD or psoriasis environment was analyzed using Affymetrix technology, covering almost all the human transcriptome (Figure 1). In the representative experiment reported, among more than 20 000 individual genes analyzed, about 750–800 genes were down- or upregulated by more than 2-fold by each treatment. These results are indicative; it should be noted that this model is a “picture” of the transcriptome of these keratinocytes under defined culture conditions, at a given time (24 h); many additional genes should have been detected under additional experimental conditions. Taking into account these remarks, we expected from these models a representative approach of a global physiopathological situation.

**Figure 2:**
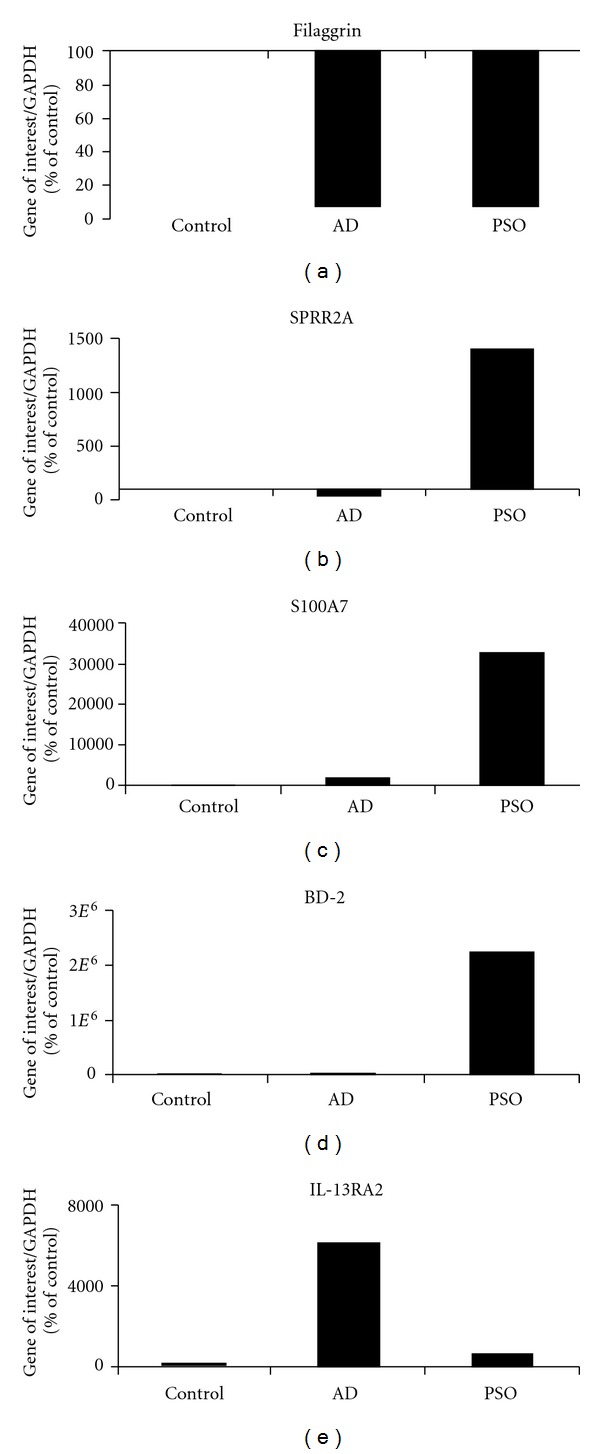
RT-qPCR analysis of the transcript expression of the keratinocyte differentiation markers filaggrin and SPRR2A, of the antimicrobial peptides S100A7 and BD-2, and the IL-13RA2 receptor chain after 24 h NHEK treatment by AD or PSO mixes. Results are expressed as percentage of the relative expression of stimulated cells over control cells.

Interestingly, there is a large overlap of more than 60% between the two treatments. Among these modulated genes, are many epidermis differentiation-related genes, such as filaggrin (Figure 2), which is known to be strongly downregulated by IL-22 (present in the two cytokine mixtures), alone or in combinations [14]. Most of the so-called epidermis differentiation markers are downregulated in NHEK by both AD and PSO NHEK treatments, as observed in pathologic skin. However, some of these markers such as genes from the small prolin rich proteins (SPRRs) family are differentially expressed in AD and PSO conditions. For example, SPRR2A is strongly overexpressed in the IL-17-containing mix (PSO) and downregulated in the IL-4/IL-13-containing mix (AD) (Figure 2).

In addition to differentiation markers, many other genes are commonly modulated by both cytokine mixes and TNF*α* by itself also strongly contribute to the “common” stimulation through. On the other hand, great differences between the two stimuli are observed for the antimicrobial peptides S100A7/psoriasin and BD-2; (Figure 2). S100A7 is dramatically overexpressed by the IL-17-containing mix (PSO) when compared to the weak enhancement induced by the IL-4/IL-13-containing mix (AD). According to its background expression in nonstimulated NHEK, BD-2 is dramatically enhanced in PSO model, and almost unmodified following AD stimulation. In contrast, the IL-13RA2 is overexpressed in the IL-4/IL-13-containing mix (AD) (Figure 2). These results parallel the *in vivo* situation, in which psoriatic skin is characterized by high expression levels of these antimicrobial peptides, functionally associated to a large degree of resistance to infections in psoriasis, and exacerbated infection sensitivity in AD skin [3].

A large number of chemokines are also produced, as indicated in Table 1. Whereas a chemokine such as CCL20 expression is more induced by the PSO mix as expected, CXCL10 is overexpressed only by the AD mix.

**Figure 3:**
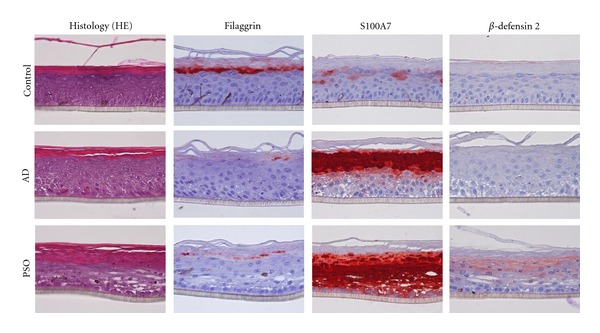
Histological and immunohistochemical analysis of RHE either unstimulated or stimulated with the IL-22, TNF*α*, IL-4, and IL-13 mixture (AD) or IL-17, IL-22, and TNF*α* mixture (PSO) for 48 h. Individual cytokine concentration was 3 ng/mL. RHE were cultured for 10 days at air-liquid interface before treatment. Tissues were fixed and embedded in paraffin and 4-*μ*m vertical sections were stained with hematoxylin-eosin or immunolabeled using antifilaggrin, anti-S100A7, or anti-hBD2 mAbs.

Since NHEK are undifferentiated primary keratinocytes cultured as monolayers, we further transpose the study to a more relevant epidermis model, reconstructed human epidermis (RHE), which closely resembles human skin epidermis (Figure 3). RHE are cornified 3D epithelia emerging from NHEK cultured at the air-medium interface [71]. The treatment of RHE with DA mix for 2 days led to significant modification of the histology of the epidermis, with an apparent loss of cohesion of the tissue, and orthokeratosis. The replacement of the Th2 cytokines by IL-17 (PSO model) leads to more dramatic tissue damage with destabilization of the epidermis, small pycnotic cells associated to parakeratosis, and a tendency to the disappearance of the granular layer. These *in vitro* phenotypes, respectively, parallel histology of AD and psoriasis lesions [3]. The decrease of expression of adhesion molecules could account for this phenotype. Clearly expressed in the epidermal layer of control RHE (Figure 3) as in normal skin, the treatment for only 48 h of these tissues by the two cytokine mixes resulted in a clear decrease in filaggrin accumulation. We previously showed that IL-22, present in the 2 mixes, decrease filaggrin expression [6]. As this effect was stronger in the AD mix, we suggested that it is due to an additive effect of IL-4/IL-13.

**Figure 4:**
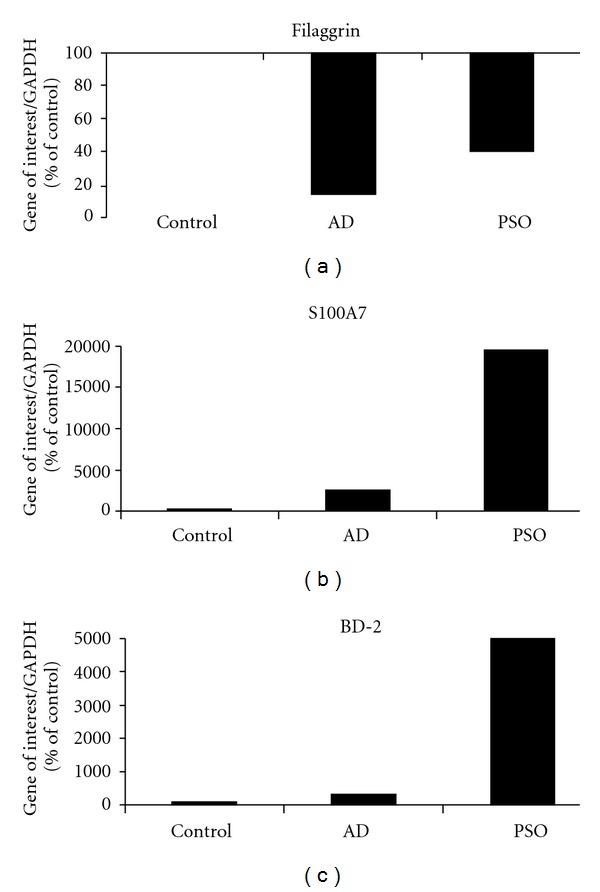
RT-qPCR analysis of the expression of the transcripts for filaggrin, S100A7, and BD-2 after 24 h treatment of RHE by AD or PSO mixes, as in Figure 3. Analyses were performed as in Figure 2.

Regarding the antimicrobial peptides, we observed a local expression of S100A7 in untreated RHE, a strong overexpression following AD treatment and, as expected, a dramatic expression with the PSO mix. The BD-2 protein is neither detected in “healthy” RHE nor in the AD model, but was strongly induced by the PSO mix. This huge enhancement results from the synergistic effect of IL-17 added to the IL-22/TNF*α* combination. We demonstrated that the immununohistological variations of filaggrin, S100A7, and BD-2 are confirmed at the mRNA level (Figure 4).

Taken together, these different patterns of *in vitro* expression were also found in chronic AD and psoriatic skin lesions. Interestingly, we demonstrate that by using exclusively keratinocytes and sets of cytokines, in the absence of other epidermis or dermis skin cells, resident or infiltrating immune cells, we can offer in vitro models that approach AD and psoriasis skin lesions. Obviously, this is downstream events that we model with keratinocytes and cytokines, depending of upstream mechanisms of recruitment and activation of other innate adaptive immune cells. Anyway, cytokine are sufficient to induce phenotypic features of chronic AD and psoriasis on keratinocytes. If still necessary, it highlights the fact that specific cytokines or combinations of cytokines are key targets for biotherapies. This approach is already largely developed for psoriasis treatments, not so far for AD treatments. Whatever, the effect of combination of cytokine are complex to analyze, and also depends on other parameters such as dose or kinetic of expression. Of course these simple keratinocyte models can be improved, but however, one lesson from these experiments is that on a common background, the presence of one additional cytokine can shift the tissue response toward a given phenotype, mutually exclusive such as in AD and psoriasis.

## 5. Concluding Remarks 

In recent years, an increasing number of reports have led to define a panel of proinflammatory cytokines able to play a central role in the induction and maintenance of chronic skin inflammation. The presence of specific subsets of Th lymphocytes polarizes the response, and keratinocyte are downstream targets of these cytokines. This cytokine environment plays a central role on the skin morphology and innate immunity, giving to keratinocytes a status of bona fide actors of the innate immunity. 

The specific cytokine environment in chronic AD includes an IL-22, TNF*α*, IL-4, and IL-13 specific combination. Altogether, these cytokines are able to mimic an “atopic dermatitis like” state on NHEK and RHE. IL-22 is especially involved in the development of the epidermal hyperplasia and hypogranulosis. TNF*α* is especially involved in the induction of the innate response, in synergy with IL-22, whereas IL-4 and IL-13 inhibits the BD-2 production. A large number of chemokines are also produced. Despite the production of neutrophil-attractive chemokines, the absence of neutrophils in AD lesions remains to be further studied. In psoriasis lesions, over expression of IL-17 is observed whereas IL-4 and IL-13 were not detected. Interestingly, the replacement of IL-4 and IL-13 by IL-17 from this cocktail is able to mimic in vitro a “psoriasis-like” status on keratinocytes. 

These studies open the way to new therapeutic strategies focusing on more specific and downstream targets, that is, keratinocytes targeting cytokines rather than on a systemic inhibition of T lymphocytes overproducing cytokines. The specific blockade of new cytokines or their receptors is an alternative approach for the treatment of AD. In any case, cytokines are very potent factors, with the advantages (or the defects, depending on the case) to be pleiotropic and redundant, and to be involved in cascade. Such treatment could reduce the biological effects of an overproduced cytokine and/or disrupt the vicious circle involving it. Targeting more than one cytokine could be a valuable strategy to assure a complete and sustained clinical improvement.

Obviously, the *in vitro* models of inflammatory epidermis induced by a specific set of cytokines could be useful tools to screen new drugs.

## Figures and Tables

**Table 1:** RT-qPCR analysis of the expression of the transcripts for chemokines. Results are expressed as the fold increase expression of stimulated cells over control cells.

Gene	Fold increase in NHEK-PSO mix	Fold increase in NHEK-AD mix
CCL5	24	43
CCL20	30	7
CCL27	11	16
CXCL1	31	16
CXCL2	4	1
CXCL5	3	1
CXCL6	4	3
CXCL8	47	37
CXCL10	1	3
